# K^+^ Efflux and Retention in Response to NaCl Stress Do Not Predict Salt Tolerance in Contrasting Genotypes of Rice (*Oryza sativa* L.)

**DOI:** 10.1371/journal.pone.0057767

**Published:** 2013-02-27

**Authors:** Devrim Coskun, Dev T. Britto, Yuel-Kai Jean, Imtiaz Kabir, Inci Tolay, Ayfer A. Torun, Herbert J. Kronzucker

**Affiliations:** 1 Department of Biological Sciences, University of Toronto, Toronto, Ontario, Canada; 2 Department of Agriculture, Akdeniz University, Antalya, Turkey; 3 Department of Soil Science & Plant Nutrition, Cukurova University, Adana, Turkey; New Mexico State University, United States of America

## Abstract

Sudden elevations in external sodium chloride (NaCl) accelerate potassium (K^+^) efflux across the plasma membrane of plant root cells. It has been proposed that the extent of this acceleration can predict salt tolerance among contrasting cultivars. However, this proposal has not been considered in the context of plant nutritional history, nor has it been explored in rice (*Oryza sativa* L.), which stands among the world’s most important and salt-sensitive crop species. Using efflux analysis with ^42^K, coupled with growth and tissue K^+^ analyses, we examined the short- and long-term effects of NaCl exposure to plant performance within a nutritional matrix that significantly altered tissue-K^+^ set points in three rice cultivars that differ in salt tolerance: IR29 (sensitive), IR72 (moderate), and Pokkali (tolerant). We show that total short-term K^+^ release from roots in response to NaCl stress is small (no more than 26% over 45 min) in rice. Despite strong varietal differences, the extent of efflux is shown to be a poor predictor of plant performance on long-term NaCl stress. In fact, no measure of K^+^ status was found to correlate with plant performance among cultivars either in the presence or absence of NaCl stress. By contrast, shoot Na^+^ accumulation showed the strongest correlation (a negative one) with biomass, under long-term salinity. Pharmacological evidence suggests that NaCl-induced K^+^ efflux is a result of membrane disintegrity, possibly as result of osmotic shock, and not due to ion-channel mediation. Taken together, we conclude that, in rice, K^+^ status (including efflux) is a poor predictor of salt tolerance and overall plant performance and, instead, shoot Na^+^ accumulation is the key factor in performance decline on NaCl stress.

## Introduction

Soil salinity, predominantly in the form of NaCl, is a major agricultural issue, particularly in irrigated areas [1], [2], where as much as one third of the world’s food production takes place and nearly half of the land is afflicted ([3] and references therein). In plants, one of the major consequences of salinity stress is a disruption in cellular and whole-plant K^+^ homeostasis [4]–[7]. Potassium is critical to the proper functioning of plant cells for reasons that include charge balancing in the cytoplasm, enzyme activation, and the maintenance of cell turgor [8], [9]. Importantly, Na^+^ has been shown to disturb the transport processes of K^+^ across the plasma membrane, specifically in root epidermal and cortical cells where Na^+^ is first encountered, by inhibiting the primary uptake of K^+^ as well as stimulating its cellular release [10]–[14].

The phenomenon of NaCl-stimulated K^+^ efflux in roots has been of much recent interest, and some controversy exists regarding its underlying mechanism. Some reports have described the effect as predominantly a channel-mediated phenomenon, where it is postulated that membrane depolarization due to Na^+^ entry (possibly via non-selective cation channels (NSCCs)) results in the opening of voltage-gated, outward-rectifying K^+^ channels [13]. An alternative explanation is that high amounts of NaCl compromises the integrity of the plasma membrane, due to ionic and osmotic effects, resulting in release of cellular contents, including K^+^ [12], [15], [16]. Understanding this phenomenon would provide important insight into uncovering the elusive nature of salt toxicity [5], [17], and would allow for critical assessment of the relevance of stimulated K^+^ efflux to other aspects of salt stress, such as the inhibition of primary K^+^ uptake, cytosolic K^+^:Na^+^ ratios, primary Na^+^ uptake, and shoot Na^+^ accumulation [4], [5], [12], [18], [19].

The development of salt-tolerant genotypes to meet increasing global food demands relies on effective and efficient screening methods for salt tolerance among crops [20]–[22]. Recently, it has been proposed that assaying NaCl-stimulated K^+^ efflux in seedling roots can be one such method, as negative correlations in barley and wheat were found between the magnitude of K^+^ efflux and physiological measures/yield data in mature plants used to identify salt tolerance [20], [23], [24]. This proposal, however, has not been explored in the chief crop species, rice (*Oryza sativa* L.), which ranks among the most salt-sensitive crops [18], [21], [25]–[27]. Furthermore, it has not been considered in the context of the nutritional conditions under which the plants have been reared. Studies on the effects of nitrogen (N) source (*i.e.*, ammonium (NH_4_^+^) vs. nitrate (NO_3_^−^)) have reported greater sensitivity of crops to salinity when NH_4_^+^ was the sole nitrogen form supplied [28]–[31]. By contrast, others have shown salinity effects to be independent of N source [32], or have reported greater sensitivity when NO_3_^−^ was the sole N source [33]. Moreover, it has been shown that K^+^ fluxes and cellular compartmentation can depend significantly on external N source and strength [34], [35]. Lastly, the application of exogenous K^+^ to alleviate plants from salinity stress is well documented [36]–[39]. Thus, it is conceivable that the extent of NaCl-stimulated K^+^ efflux can differ significantly depending on growth history, particularly with respect to K^+^ and N nutrition, and should be critically considered before broader conclusions are drawn regarding the utility of such a screening tool.

In the present study, we tested the hypothesis that the extent of K^+^ efflux upon short-term exposure to NaCl can predict plant performance on long-term NaCl stress in three cultivars of rice (*Oryza sativa* L.) that differ in salt sensitivity: IR29 (sensitive), IR72 (moderate), and Pokkali (tolerant). Plants were grown under eight nutritional regimes varying in N source (NH_4_^+^ vs. NO_3_^−^), N strength (0.1 vs. 10 mM), and K^+^ strength (0.1 vs. 1.5 mM), to investigate the effects of these two key macronutrients to K^+^ status and growth, in relation to performance on short- and long-term NaCl stress. Responses to short-term NaCl stress that were considered include: (1) peak NaCl-stimulated K^+^ efflux, (2) cytosolic K^+^ release, and (3) total root K^+^ loss. Measures of long-term NaCl stress include: (1) survival, (2) biomass, (3) tissue K^+^ content, and (4) tissue Na^+^ content. We show that, surprisingly, no measure of K^+^ fluxes or accumulation could predict plant performance in the presence *or* absence of NaCl stress, and that instead, shoot Na^+^ content was the best indicator of performance on high salinity, albeit after the fact.

## Materials and Methods

### Plant material and growth conditions

Rice seeds (*Oryza sativa* L., cvs. ‘IR29’, ‘IR72’, and ‘Pokkali’) were surface-sterilized with 1% (v/v) sodium hypochlorite for 10 min, germinated in aerated dH_2_O for 48 h, and placed into 14-L plastic hydroponic vessels containing aerated, modified Johnson’s solutions (2 mM MgSO_4_, 0.3 mM NaH_2_PO_4_, 0.3 mM CaCl_2_, 0.1 mM Fe-EDTA, 20 µM H_3_BO_3_, 9 µM MnCl_2_, 1.5 µM CuSO_4_, 1.5 µM ZnSO_4_, 0.5 µM Na_2_MoO_4_), pH 6.30–6.35 (adjusted with 1 M NaOH). Potassium was supplied as K_2_SO_4_ at either 0.1 or 1.5 mM. Nitrogen was supplied either as Ca(NO_3_)_2_ or (NH_4_)_2_SO_4_, at either 0.1 or 10 mM N. Long-term salinity stress treatments involved supplementation of the growth medium with 50 mM NaCl. To ensure plants were maintained at a nutritional steady state, solutions were completely exchanged on days 9, 13, 16, 18, and 20 (post-sterilization), and were experimented with on day 21. Plants were cultured in climate-controlled, walk-in growth chambers under fluorescent lights with an irradiation of 425 µmol photons m^−2^ s^−1^ at plant height for 12 h d^−1^ (Philips Silhouette High Output F54T5/850HO; Philips Electronics Ltd, Markham, ON, Canada). Day/night temperature cycle was 30°C/20°C, and relative humidity was 70%.

### Tissue K^+^ and Na^+^ content

The measurement of tissue K^+^ and Na^+^ content was performed as previously described [12], [19]. In brief, roots of intact 21-d-old seedlings were desorbed in aerated 10 mM CaSO_4_ for 5 min, to release extracellular K^+^ (steady-state conditions). For a subset of seedlings grown without NaCl stress, roots were first immersed in aerated growth solution supplemented with 160 mM NaCl for 45 min (to parallel efflux experiments, see below), followed by desorption in 10 mM CaSO_4_ for 5 min (short-term NaCl treatment). From there, roots were detached from shoots and spun in a low-speed centrifuge for 30 s, to remove surface water. After weighing, tissue samples were oven-dried for three days at 85 – 90°C, and then pulverized (VWR VDI12 homogenizer; VWR International, Mississauga, ON, Canada) and digested for an additional three days in 30% (v/v) HNO_3_. K^+^ and Na^+^ concentrations of the tissue digests were quantified using a dual-channel flame photometer (Model 2655-10; Cole-Parmer Instrument Company, Anjou, QC, Canada).

### ^42^K^+^ efflux

Two days prior to experimentation (day 19), seedlings were bundled together in groups of five at the stem base by a 0.5-cm-high plastic collar. Only plants grown without NaCl were used to monitor changes in potassium efflux over time due to sudden NaCl exposure. ^42^K^+^ efflux from roots of intact seedlings was monitored as described previously [12], [34], [40] and based on the method from compartmental analysis [41]–[43]. In brief, roots were immersed for 1 h in a nutrient solution identical to growth conditions but containing ^42^K (*t_1/2_*  =  12.36 h), received as ^42^K_2_CO_3_ from the McMaster University Nuclear Reactor (Hamilton, ON, Canada). From there, seedlings were secured into glass efflux funnels and roots were eluted of radioactivity with successive 20-mL aliquots on non-radioactive growth solution. The desorption series was timed as follows, from first to final eluate: 15 s (four times), 20 s (three times), 30 s (twice), 40 s (once), 50 s (once), 1 min (23 times), 1.5 min (three times), 2 min (three times), 3 min (three times), 4 min (twice), and 5 min (once), for a total of 1 h of elution. The first 22 eluates (15.5 min into the desorption series) were identical to growth solution and the final 24 eluates contained growth solution supplemented with 160 mM NaCl. A subset of experiments involved the final 24 eluates with 160 mM NaCl co-supplied with either 10 mM CaCl_2_ or 10 mM CsCl.

Immediately following the elution series, seedling bundles had their roots detached from shoots and centrifuged, as described above, before weighing. Radioactivity from eluates, roots, and shoots, was counted, and corrected for isotopic decay, using a gamma counter (PerkinElmer Wallac 1480 Wizard 3″; Turku, Finland). For comparison charts of ^42^K^+^ efflux, the specific activities of all replicates were normalized to an arbitrary value of 2×10^5^ cpm μmol^−1^.

### Estimation of cytosolic K^+^ release and K^+^ efflux during NaCl treatment

As previously observed in barley [12], sudden application of high (160 mM) NaCl during a ^42^K^+^ efflux protocol resulted in significant stimulations in radiotracer release from roots over the course of the treatment (see Results). To express this release in terms of μmol K^+^ g^−1^ (root fresh weight (FW)), an integration procedure was employed similar to that previously described [40]. Based on literature precedence demonstrating the extremely slow rate of vacuolar K^+^ loading relative to the cytosol (*e.g.*, several hours as compared to several minutes, respectively) [44]–[47], we could assume that after 1 h of loading, the majority of tracer accumulation occurs within the cytosol. However, since phase testing yielded no evidence for physiological efflux in our model system (data not shown; *cf*. [34], [40]), no estimates of cytosolic exchange kinetics were made [41]–[43], and specific activity of the cytosol (SA_cyt_) was estimated to be equal to external specific activity (SA_ext_). Thus, to quantify the minimum amount of K^+^ released from the cytosol (in μmol K^+^ g^−1^ (root FW)) during NaCl treatment, the radioactivity released (in cpm) during this period was summed, divided by SA_cyt_ (in cpm μmol^−1^), and corrected for root FW. This protocol was conducted for each replicate within a treatment and averaged for each individual treatment (± SEM).

Similarly, the peak magnitude of NaCl-stimulated K^+^ efflux (in μmol g^−1^ (root FW) h^−1^) was estimated for each cultivar and growth condition, by dividing the maximal rate of radioactivity release (in cpm released g^−1^ (root FW) min^−1^; this generally occurred within the first 2 min of treatment - see Results) by SA-_cyt_ and correcting for time. This protocol was used for each replicate within a treatment and then averaged for each treatment (± SEM).

### Statistics

For efflux experiments, each bundle of five seedlings was considered a single replicate, and each treatment was replicated a minimum of three times. For growth and tissue content analyses, each bundle of four seedlings was considered a single replicate, with a minimum replication of four. Comparisons in cytosolic K^+^ release and peak K^+^ efflux during NaCl treatment (as described above) were analyzed within a single variant by use of one-way ANOVA with Bonferroni *post-hoc* corrections. Student’s *t*-tests were performed to determine significantly different means in K^+^ and Na^+^ content between control and Na^+^-treated plants. Pearson correlation analyses were performed using GraphPad Prism 5 (GraphPad Software; La Jolla, CA, USA).

## Results

**Figure 1:**
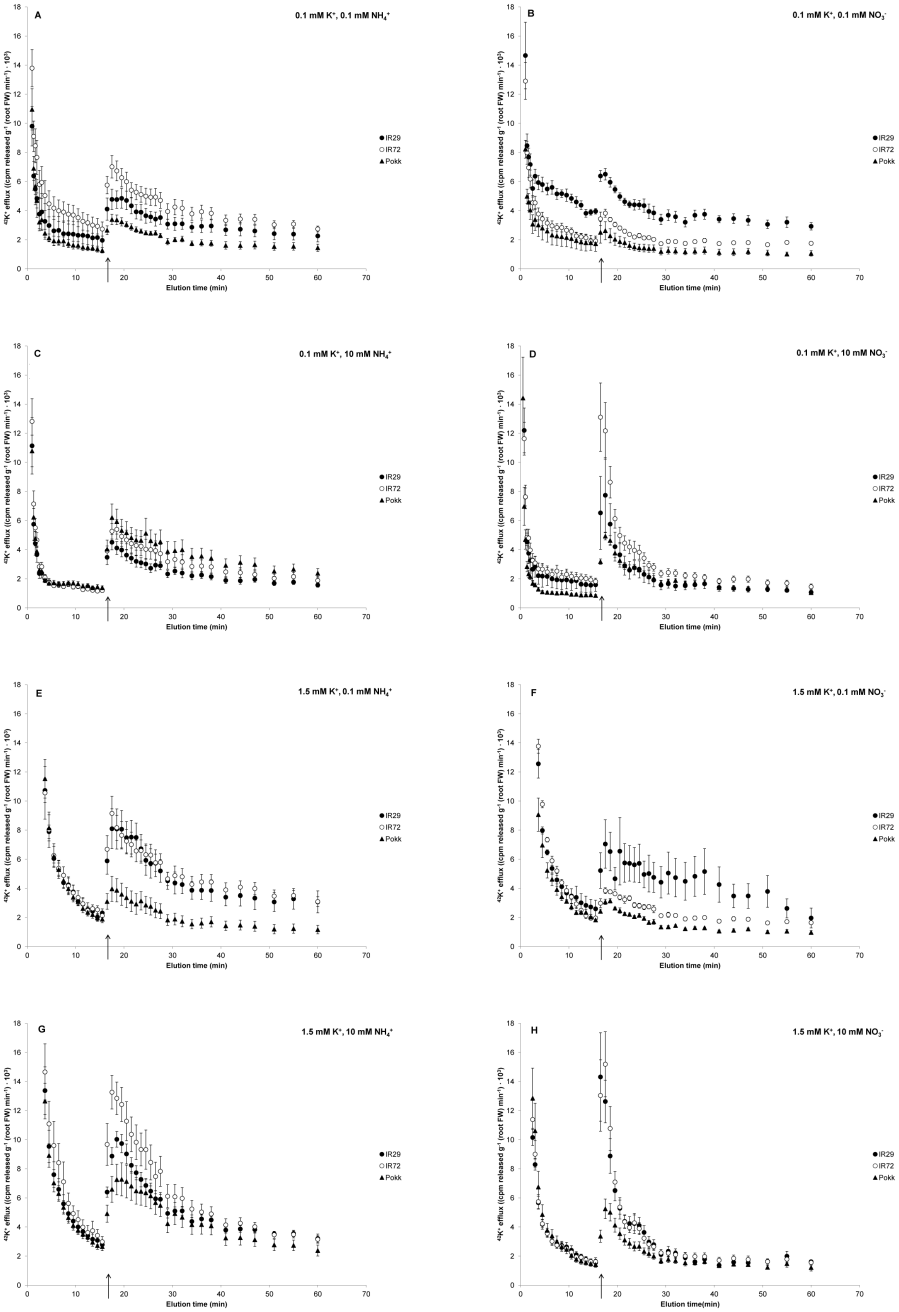
Nutritional and cultivar comparisons of NaCl-stimulated K^+^ efflux. Cultivar differences in ^42^K^+^ efflux from roots of intact rice (*Oryza sativa* L. cvs. ‘IR29’, ‘IR72’, and ‘Pokkali’) in response to sudden provision (at *t*  =  15.5 min, see arrow) of 160 mM NaCl. Seedlings were grown and tested in a full nutrient medium supplemented with either 0.1 (A – D) or 1.5 mM K^+^ (E – H), and one of four N regimes: 0.1 mM NH_4_^+^ (A, E), 0.1 mM NO_3_^−^ (B, F), 10 mM NH_4_^+^ (C, G), and 10 mM NO_3_^−^ (D, H). Error bars indicate ± SEM.

Fig. 1 shows the release kinetics of ^42^K^+^ from roots of intact, pre-labeled, rice seedlings, and their response to sudden application of 160 mM NaCl (at *t*  =  15.5 min, see arrow), in three cultivars that differ in salt tolerance: IR29 (sensitive), IR72 (moderate), and Pokkali (tolerant). Seedlings were grown and measured under eight nutritional conditions that varied in N source (NH_4_^+^ or NO_3_^−^), N strength (0.1 or 10 mM, referred to as ‘low’ and ‘high’, respectively), and K^+^ strength (0.1 or 1.5 mM, also referred to as ‘low’ and high’), which had considerable effects on plant biomass and tissue K^+^ content (Tables 1, 2, 3, 4, also see below).

**Table 1:** Long-term NaCl exposure and tissue biomass and content (low K^+^, NH_4_^+^ conditions).

	0.1 mM K^+^											
	0.1 mM NH_4_^+^						10 mM NH_4_^+^					
	IR29		IR72		Pokkali		IR29		IR72		Pokkali	
	−Na^+^	+Na^+^	−Na^+^	+Na^+^	−Na^+^	+Na^+^	−Na^+^	+Na^+^	−Na^+^	+Na^+^	−Na^+^	+Na^+^
Shoot FW^1^ (g)	0.703±0.061	—	0.786±0.057	0.328±0.023	0.988±0.051	0.746±0.061	0.251±0.037	—	0.428±0.034	—	1.133±0.036	0.573±0.042
Root FW (g)	0.393±0.049	—	0.557±0.039	0.246±0.030	0.577±0.035	0.360±0.030	0.112±0.016	—	0.169±0.018	—	0.289±0.012	0.194±0.060
Total FW (g)	1.096±0.109	—	1.342±0.096	0.574±0.052	1.565±0.083	1.106±0.091	0.363±0.052	—	0.597±0.050	—	1.421±0.047	0.767±0.050
Total FW decline (g)	—		0.768±0.109^***^		0.459±0.123^**^		—		—		0.655±0.068^***^	
Total FW decline (% Ctrl^2^)	—		57.23		29.33		—		—		46.02	
Shoot DW^3^:FW ratio	0.190±0.001	—	0.182±0.001	0.233±0.006	0.156±0.002	0.181±0.002	0.199±0.005	—	0.192±0.002	—	0.168±0.003	0.196±0.004
Root DW:FW ratio	0.082±0.004	—	0.083±0.003	0.066±0.004	0.090±0.004	0.068±0.003	0.049±0.009	—	0.063±0.003	—	0.078±0.002	0.062±0.003
SKC^4^ (μmol g^−1^ FW)	169.9±2.0	—	185.7±1.5	188.4±10.0	185.8±0.6	168.0±2.9	105.9±1.7	—	84.2±7.7	—	71.1±1.0	40.0±1.0
RKC^5^ (μmol g^−1^ FW)	73.4±0.3	—	76.4±1.4	23.2±0.5	69.2±2.1	28.7±0.8	42.8±3.1	—	24.4±1.3	—	24.2±0.3	17.9±0.6
RKC decline (μmol g^−1^ FW)	—		53.3±2.3^***^		40.5±2.2^***^		—		—		6.3±0.7^***^	
RKC decline (% Ctrl)	—		69.63		58.53		—		—		26.0	
SNaC^6^ (μmol g^−1^ FW)	12.1±0.9	—	15.0±0.9	574.6±63.5	13.2±1.4	257.8±10.8	37.6±3.7	—	34.3±6.7	—	15.3±1.0	213.9±24.6
RNaC^7^ (μmol g^−1^ FW)	15.5±1.3	—	14.4±0.9	167.4±20.3	13.5±1.6	126.8±14.4	35.8±2.6	—	31.7±3.2	—	18.9±0.9	59.8±3.5

Steady-state biomass and tissue content values of three rice (*Oryza sativa* L.) cultivars grown

under low K^+^ and NH_4_^+^ conditions, +/−50 mM NaCl. Dashes indicate instances of mortality. Asterisks denote different levels of significance between control and treatment pairs (ns: not significant, *: 0.01<P<0.05, **: 0.001<P<0.01, ***: P<0.001; Student’s *t*-test).

1 Fresh weight; ^2^Control; ^3^Dry weight; ^4^Shoot K^+^ content; ^5^Root K^+^ content; ^6^Shoot Na^+^ content; ^7^Root Na^+^ content.

**Table 2:** Long-term NaCl exposure and tissue biomass and content (low K^+^, NO_3_^−^ conditions).

	0.1 mM K^+^											
	0.1 mM NO_3_^−^						10 mM NO_3_^−^					
	IR29		IR72		Pokkali		IR29		IR72		Pokkali	
	−Na^+^	+Na^+^	−Na^+^	+Na^+^	−Na^+^	+Na^+^	−Na^+^	+Na^+^	−Na^+^	+Na^+^	−Na^+^	+Na^+^
Shoot FW^1^ (g)	0.866±0.036	—	0.864±0.044	0.214±0.031	1.363±0.126	0.582±0.026	0.832±0.057	0.341±0.019	0.888±0.052	0.456±0.026	1.757±0.043	0.808±0.066
Root FW (g)	0.610±0.034	—	0.754±0.025	0.168±0.027	1.047±0.055	0.362±0.014	0.449±0.098	0.253±0.011	0.424±0.026	0.291±0.022	0.615±0.022	0.416±0.031
Total FW (g)	1.476±0.062	—	1.618±0.068	0.382±0.058	2.410±0.176	0.944±0.039	1.281±0.137	0.594±0.022	1.312±0.078	0.747±0.047	2.372±0.063	1.225±0.095
Total FW decline (g)	—		1.236±0.090^***^		1.466±0.181^***^		0.687±0.164^**^		0.565±0.091^***^		1.147±±0.114^***^	
Total FW decline (% Ctrl^2^)	—		76.39		60.83		53.63		43.06		48.36	
Shoot DW^3^:FW ratio	0.177±0.002	—	0.175±0.002	0.229±0.009	0.173±0.002	0.198±0.003	0.195±0.001	0.263±0.009	0.179±0.001	0.230±0.002	0.157±0.003	0.193±0.003
Root DW:FW ratio	0.084±0.001	—	0.088±0.002	0.058±0.004	0.100±0.001	0.070±0.002	0.099±0.014	0.073±0.001	0.079±0.001	0.075±0.001	0.082±0.001	0.073±0.001
SKC^4^ (μmol g^−1^ FW)	199.8±1.4	—	177.9±1.7	188.1±25.1	179.1±3.4	120.0±3.2	173.2±1.4	125.2±7.9	176.0±2.6	129.5±3.5	180.8±8.4	129.7±6.0
RKC^5^ (μmol g^−1^ FW)	92.7±1.2	—	85.7±1.6	33.4±3.5	81.3±4.7	27.6±1.2	55.2±5.0	23.1±0.8	47.3±3.0	30.8±0.4	50.2±1.4	26.6±1.1
RKC decline (μmol g^−1^ FW)	—		52.3±3.4^***^		53.7±4.8^***^		32.1±5.1^**^		16.5±4.8^**^		23.6±1.7^***^	
RKC decline (% Ctrl)	—		61.03		66.05		58.15		34.88		47.01	
SNaC^6^ (μmol g^−1^ FW)	27.1±5.4	—	16.1±1.3	583.2±138.8	14.5±1.4	297.5±29.3	14.5±0.8	382.4±37.2	16.9±3.3	238.6±16.6	15.8±5.1	124.9±18.0
RNaC^7^ (μmol g^−1^ FW)	16.3±1.2	—	14.4±0.5	117.6±7.6	16.2±0.7	107.6±2.2	7.9±0.2	66.0±1.5	11.2±0.9	64.0±1.0	15.3±0.4	58.7±2.2

Steady-state biomass and tissue content values of three rice (*Oryza sativa* L.) cultivars grown under low K^+^ and NO_3_^−^ conditions, +/−50 mM NaCl. Dashes indicate instances of mortality. Asterisks denote different levels of significance between control and treatment pairs (ns: not significant, *: 0.01<P<0.05, **: 0.001<P<0.01, ***: P<0.001; Student’s *t*-test).

1 Fresh weight; ^2^Control; ^3^Dry weight; ^4^Shoot K^+^ content; ^5^Root K^+^ content; ^6^Shoot Na^+^ content; ^7^Root Na^+^ content.

**Table 3:** Long-term NaCl exposure and tissue biomass and content (high K^+^, NH_4_^+^ conditions).

	1.5 mM K^+^											
	0.1 mM NH_4_^+^						10 mM NH_4_^+^					
	IR29		IR72		Pokkali		IR29		IR72		Pokkali	
	−Na^+^	+Na^+^	−Na^+^	+Na^+^	−Na^+^	+Na^+^	−Na^+^	+Na^+^	−Na^+^	+Na^+^	−Na^+^	+Na^+^
Shoot FW^1^ (g)	0.485±0.076	—	0.937±0.104	0.215±0.052	1.120±0.125	1.015±0.044	0.688±0.083	—	0.743±0.062	0.254±0.038	1.908±0.111	1.071±0.057
Root FW (g)	0.248±0.048	—	0.536±0.059	0.138±0.024	0.665±0.081	0.483±0.027	0.298±0.052	—	0.317±0.024	0.140±0.026	0.640±0.031	0.365±0.018
Total FW (g)	0.734±0.124	—	1.473±0.161	0.353±0.076	1.785±0.205	1.497±0.070	0.986±0.134	—	1.060±0.085	0.394±0.062	2.547±0.140	1.437±0.071
Total FW decline (g)	—		1.120±0.178^***^		0.287±0.217^ns^		—		0.666±0.106^***^		1.111±0.157^***^	
Total FW decline (% Ctrl^2^)	—		76.04		16.13		—		62.83		43.58	
Shoot DW^3^:FW ratio	0.184±0.001	—	0.165±0.002	0.245±0.019	0.150±0.003	0.168±0.002	0.169±0.002	—	0.165±0.001	0.197±0.008	0.154±0.006	0.161±0.001
Root DW:FW ratio	0.066±0.003	—	0.073±0.001	0.050±0.016	0.091±0.001	0.070±0.001	0.056±0.009	—	0.064±0.001	0.041±0.010	0.078±0.002	0.064±0.004
SKC^4^ (μmol g^−1^ FW)	202.3±5.1	—	194.4±3.0	244.6±34.4	205. 8±3.3	168.4±2.5	166.1±4.0	—	198.5±3.4	172.3±10.9	202.5±6.8	122.1±2.2
RKC^5^ (μmol g^−1^ FW)	105.4±1.8	—	105.0±3.5	41.6±3.4	112.2±2.1	51.5±0.9	61.4±1.6	—	64.5±1.5	27.2±0.8	70.8±1.4	32.8±0.6
RKC decline (μmol g^−1^ FW)	—		63.5±6.2^***^		60.7±4.0^***^		—		37.3±2.5^***^		38.0±2.3^***^	
RKC decline (% Ctrl)	—		60.38		54.10		—		57.83		53.67	
SNaC^6^ (μmol g^−1^ FW)	17.7±3.6	—	13.7±1.6	642.8±137.4	11.9±1.5	77.8±6.9	25.9±3.8	—	23.9±3.7	382.1±73.4	17.3±0.9	130.0±15.6
RNaC^7^ (μmol g^−1^ FW)	11.8±2.1	—	11.2±1.0	112.4±6.4	11.8±0.7	86.7±1.4	15.9±3.9	—	16.4±2.3	128.6±25.0	10.0±0.4	67.2±2.5

Steady-state biomass and tissue content values of three rice (*Oryza sativa* L.) cultivars grown under high K^+^ and NH_4_^+^ conditions, +/−50 mM NaCl. Dashes indicate instances of mortality. Asterisks denote different levels of significance between control and treatment pairs (ns: not significant, *: 0.01<P<0.05, **: 0.001<P<0.01, ***: P<0.001; Student’s *t*-test).

1 Fresh weight; ^2^Control; ^3^Dry weight; ^4^Shoot K^+^ content; ^5^Root K^+^ content; ^6^Shoot Na^+^ content; ^7^Root Na^+^ content.

**Table 4:** Long-term NaCl exposure and tissue biomass and content (high K^+^, NO_3_^−^ conditions).

	1.5 mM K^+^											
	0.1 mM NO_3_^−^						10 mM NO_3_^−^					
	IR29		IR72		Pokkali		IR29		IR72		Pokkali	
	−Na^+^	+Na^+^	−Na^+^	+Na^+^	−Na^+^	+Na^+^	−Na^+^	+Na^+^	−Na^+^	+Na^+^	−Na^+^	+Na^+^
Shoot FW^1^ (g)	0.687±0.095	—	0.798±0.058	—	1.425±0.113	1.029±0.057	0.814±0.065	—	0.969±0.067	0.246±0.047	2.220±0.129	1.120±0.080
Root FW (g)	0.462±0.069	—	0.487±0.044	—	0.928±0.098	0.572±0.023	0.348±0.030	—	0.480±0.043	0.155±0.028	0.834±0.023	0.473±0.025
Total FW (g)	1.149±0.135	—	1.285±0.099	—	2.352±0.201	1.601±0.076	1.162±0.094	—	1.449±0.107	0.401±0.075	3.053±0.140	1.593±0.100
Total FW decline (g)	—		—		0.751±0.215^*^		—		1.048±0.131^***^		1.460±0.172^***^	
Total FW decline (% Ctrl^2^)	—		—		31.93		—		72.33		47.82	
Shoot DW^3^:FW ratio	0.166±0.003	—	0.170±0.003	—	0.158±0.001	0.158±0.001	0.170±0.002	—	0.173±0.002	0.233±0.017	0.152±0.001	0.178±0.001
Root DW:FW ratio	0.070±0.002	—	0.074±0.002	—	0.091±0.001	0.072±0.008	0.070±0.001	—	0.069±0.002	0.039±0.005	0.076±0.001	0.070±0.002
SKC^4^ (μmol g^−1^ FW)	230.3±6.5	—	202.7±4.6	—	202.1±2.9	157.9±4.7	184.8±2.8	—	174.3±1.2	218.8±32.6	207.1±5.4	137.2±3.8
RKC^5^ (μmol g^−1^ FW)	100.9±4.0	—	99.6±3.2	—	99.5±4.2	46.7±1.4	83.3±2.6	—	77.5±2.7	42.2±1.5	85.6±3.1	37.8±1.4
RKC decline (μmol g^−1^ FW)	—		—		52.8±6.8^***^		—		32.3±4.4^***^		47.8±5.0^***^	
RKC decline (% Ctrl)	—		—		53.07		—		45.55		55.84	
SNaC^6^ (μmol g^−1^ FW)	20.2±5.6	—	12.3±1.1	—	14.7±2.1	143.8±7.4	19.3±1.3	—	19.5±2.9	453.6±95.5	14.4±3.2	133.5±11.6
RNaC^7^ (μmol g^−1^ FW)	8.6±0.4	—	5.9±0.4	—	26.5±0.6	82.0±3.3	12.7±1.6	—	9.8±1.3	75.5±5.0	10.4±1.9	50.7±1.3

Steady-state biomass and tissue content values of three rice (*Oryza sativa* L.) cultivars grown under high K^+^ and NO_3_^−^ conditions, +/−50 mM NaCl. Dashes indicate instances of mortality. Asterisks denote different levels of significance between control and treatment pairs (ns: not significant, *: 0.01<P<0.05, **: 0.001<P<0.01, ***: P<0.001; Student’s *t*-test).

1 Fresh weight; ^2^Control; ^3^Dry weight; ^4^Shoot K^+^ content; ^5^Root K^+^ content; ^6^Shoot Na^+^ content; ^7^Root Na^+^ content.

As was previously shown in barley [12], sudden exposure of roots to 160 mM NaCl caused an immediate stimulation of ^42^K^+^ efflux in rice seedlings. This response was observed in all cultivars, regardless of growth condition (Fig. 1). We should note, however, that this response was not observed at lower [NaCl] (*i.e.*, 25–75 mM; Fig. S1), although 50 mM NaCl was effective at suppressing growth in all three cultivars (see below). Salt-tolerant Pokkali displayed lower NaCl-stimulated K^+^ efflux, relative to the other cultivars, in terms of both peak efflux and an integration of all ^42^K^+^ released during elution (Table 5), under all growth conditions except for low K^+^, high NH_4_^+^ (Fig. 1D). By contrast, although IR72 displayed intermediate salt sensitivity (as measured by survival, biomass decline, and shoot Na^+^ content; Tables 1, 2, 3, 4), this was not generally reflected in the extent of NaCl-stimulated K^+^ efflux. In fact, only under low nitrate conditions did efflux in IR72 fall between that of IR29 and Pokkali (Fig. 1B & F).

**Table 5:** Short-term NaCl exposure and K^+^ efflux and retention.

	0.1 mM K^+^											
	0.1 mM NH_4_^+^			10 mM NH_4_^+^			0.1 mM NO_3_^−^			10 mM NO_3_^−^		
	IR29	IR72	Pokk	IR29	IR72	Pokk	IR29	IR72	Pokk	IR29	IR72	Pokk
peak efflux (μmol g^−1^ h^−1^)	0.026±0.004^ab^	0.037±0.004^b^	0.018±0.001^a^	0.023±0.003^a^	0.028±0.003^a^	0.032±0.005^a^	0.034±0.002^c^	0.017±0.001^b^	0.010±0.002^a^	0.049±0.014^ab^	0.073±0.011^b^	0.025±0.001^a^
K^+^_cyt_ 1 loss (μmol g^−1^)	0.677±0.096^ab^	0.886±0.084^b^	0.435±0.048^a^	0.519±0.049^a^	0.660±0.089^a^	0.789±0.117^a^	0.898±0.083^c^	0.430±0.009^b^	0.227±0.041^a^	0.441±0.081^a^	0.667±0.075^a^	0.419±0.019^a^
K^+^_tissue_^2^ loss (μmol g^−1^)	1.224±0.992^ns^	3.371±1.769^ns^	−8.911±3.859^ns^	n.d.^3^	7.100±1.508^***^	7.547±0.941^***^	10.690±3.038^*^	7.185±2.368^**^	−4.409±7.129^ns^	3.063±4.270^ns^	3.738±3.627^ns^	9.165±1.704^**^

	1.5 mM K^+^											
	0.1 mM NH_4_^+^			10 mM NH_4_^+^			0.1 mM NO_3_^−^			10 mM NO_3_^−^		
	IR29	IR72	Pokk	IR29	IR72	Pokk	IR29	IR72	Pokk	IR29	IR72	Pokk
peak efflux (μmol g^−1^ h^−1^)	0.042±0.007^b^	0.046±0.006^b^	0.021±0.004^a^	0.052±0.003^ab^	0.069±0.012^b^	0.039±0.006^a^	0.035±0.008^b^	0.020±0.001^a^	0.016±0.001^a^	0.077±0.013^b^	0.076±0.011^b^	0.027±0.003^a^
K^+^_cyt_ loss (μmol g^−1^)	0.980±0.171^b^	1.051±0.126^b^	0.452±0.096^a^	1.102±0.079^a^	1.426±0.201^a^	0.950±0.135^a^	0.934±0.207^b^	0.486±0.027^a^	0.324±0.015^a^	0.571±0.045^ab^	0.678±0.083^b^	0.406±0.057^a^
K^+^_tissue_ loss (μmol g^−1^)	11.550±2.473^***^	12.720±3.363^**^	4.595±3.704^ns^	7.162±2.522^*^	7.706±2.241^**^	4.309±2.943^ns^	3.912±4.594^ns^	12.950±4.034^**^	10.170±6.186^ns^	14.270±6.424^*^	19.890±4.284^**^	15.530±7.379^*^

Effect of sudden application of 160 mM NaCl to K^+^ efflux and K^+^ content loss (from the cytosol and total tissue) from roots of three rice (*Oryza sativa* L.) cultivars grown under varying nutritional conditions. Letters indicate significantly different means (one-way ANOVA with Bonferroni post-test, P<0.05) between cultivars within an N regime. Asterisks denote different levels of significance between control and treatment pairs used to determine K^+^_tissue_ loss (ns: not significant, *: 0.01<P<0.05, **: 0.001<P<0.01, ***: P<0.001; Student’s *t*-test).

1 cytosolic K^+^; ^2^total tissue K^+^; ^3^not determined.

**Figure 2:**
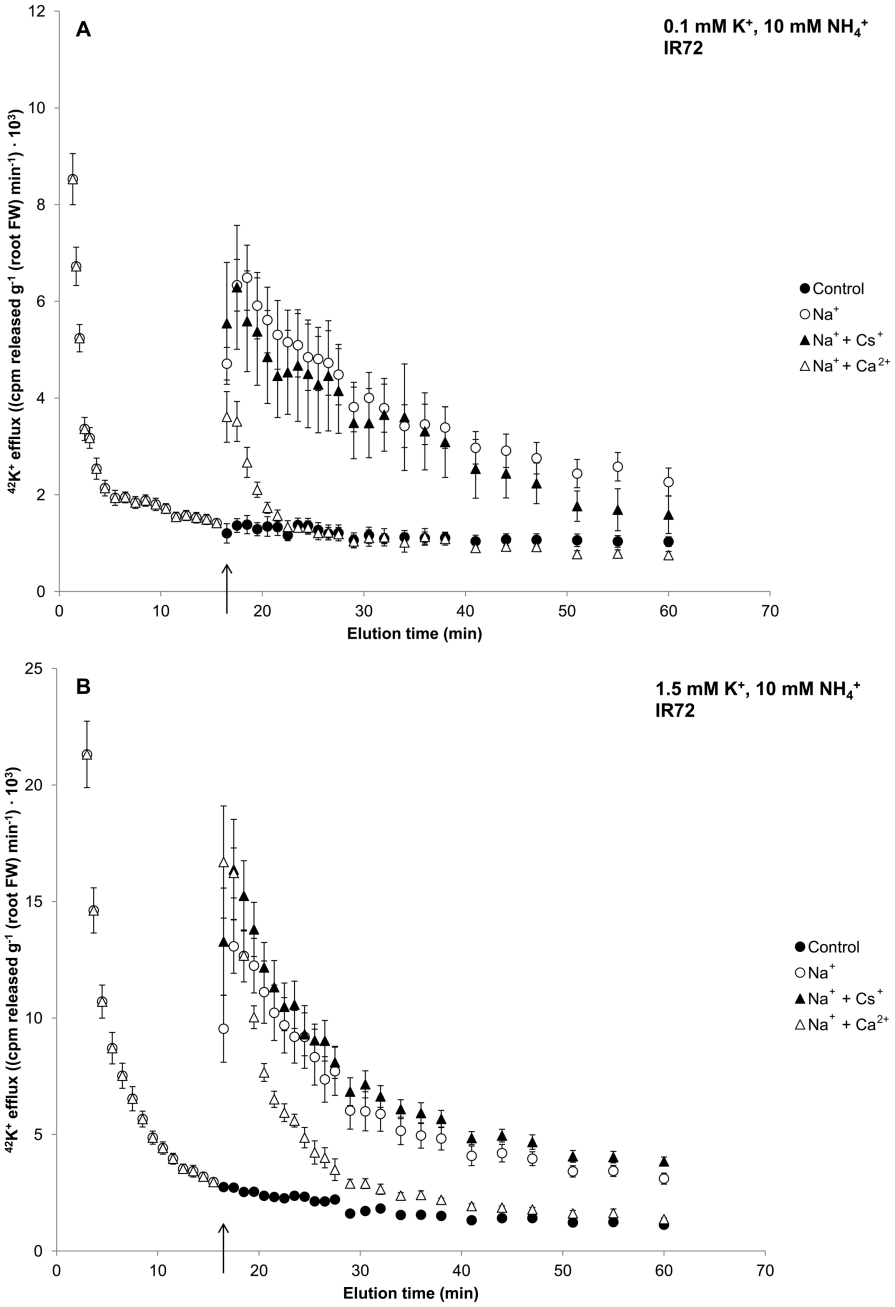
Inhibitor effects of NaCl-stimulated K^+^ efflux. The effect of co-application of 10 mM CsCl or CaCl_2_ with sudden provision (at *t*  =  15.5 min, see arrow) of 160 mM NaCl on the response of ^42^K^+^ efflux from roots of intact rice (*Oryza sativa* L.) in the cultivar IR72. External N source was supplied as 10 mM NH_4_^+^ and K^+^ at either 0.1 (A) or 1.5 mM (B). Error bars indicate ± SEM.

Fig. 2 illustrates the sensitivity of NaCl-stimulated K^+^ efflux in IR72 to selected ion channel inhibitors. Under the conditions tested, NaCl-stimulated K^+^ efflux showed no sensitivity to Cs^+^, a potent inhibitor of K^+^ channels, including outward-rectifying K^+^ channels [34], [48]. By contrast, NaCl-stimulated K^+^ efflux displayed significant sensitivity to added Ca^2+^, which is known to both inhibit NSCCs [49]–[51] and stabilize membranes [16], [52], [53]. This was particularly noticeable under low-K^+^ conditions (Fig. 2A).

**Figure 3:**
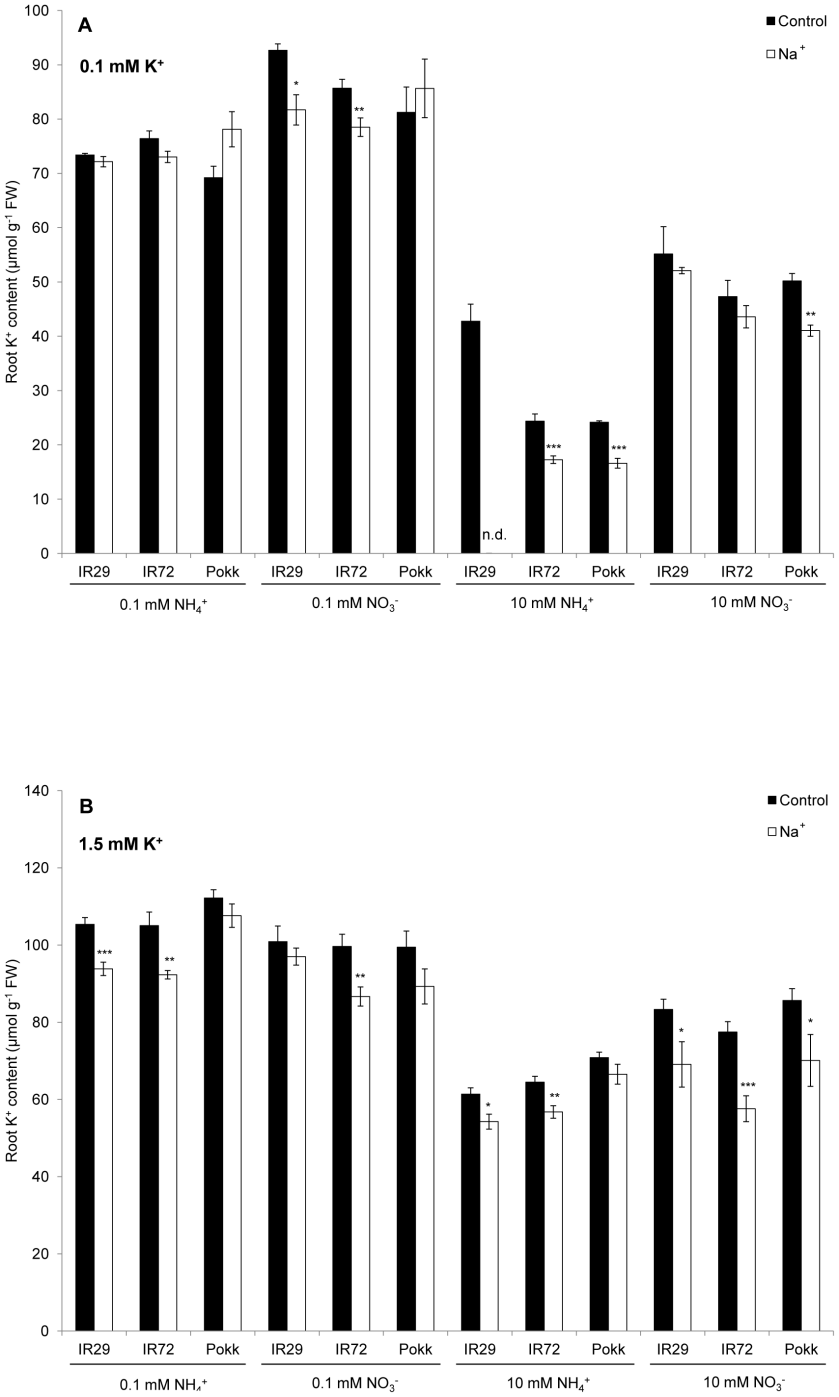
Root K^+^ content and short-term NaCl stress. Root K^+^ content, before and after short-term (45 min) exposure to 160 mM NaCl, in three cultivars of rice (*Oryza sativa* L., cvs. ‘IR29’, ‘IR72’, and ‘Pokkali’). Plants were grown and tested in a full nutrient medium supplemented with either 0.1 (A) or 1.5 mM K^+^ (B), and one of four N regimes: 0.1 mM NH_4_^+^, 0.1 mM NO_3_^−^, 10 mM NH_4_^+^, and 10 mM NO_3_^−^. Asterisks denote different levels of significance between control and treatment pairs (ns: not significant, *: 0.01<P<0.05, **: 0.001<P<0.01, ***: P<0.001; Student’s *t*-test). Error bars indicate ± SEM.

Total K^+^ content of roots before and after short-term NaCl stress (45-min exposure to 160 mM NaCl) showed relatively little decline (Fig. 3). No more than 20 µmol K^+^ g^−1^ FW were lost (see IR72 at high K^+^, high NO_3_^−^; Table 5), which amounted to a maximal decline of 26% compared to control (∼78 µmol g^−1^; Fig. 3, Table 4). These losses were considerably smaller than the differences in root K^+^ content among cultivars in the absence of NaCl stress, where amounts ranged between 24 µmol g^−1^ FW (at low K^+^, high NH_4_^+^) and 112 µmol g^−1^ FW (high K^+^, low NH_4_^+^) (Fig. 3; Table 1 and Table 3, respectively). In the presence of long-term NaCl stress, root K^+^ content ranged from 18 to 52 µmol g^−1^ FW, depending on growth history, amounting to a maximal decline of 70% compared to control (see IR72 at low K^+^, low NH_4_^+^; Table 1).

**Figure 4:**
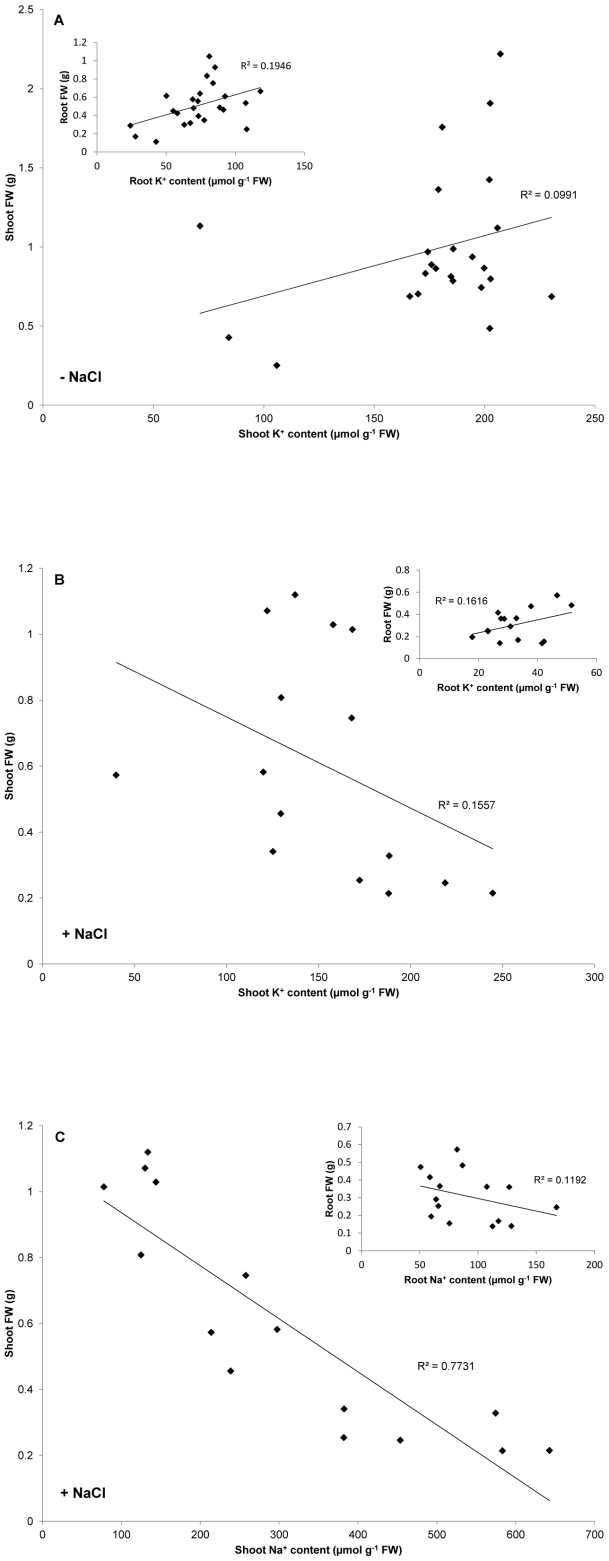
Tissue K^+^/Na^+^ content and biomass. Correlation analyses between shoot K^+^ content and fresh weight, in the absence (A) or presence (B) of long-term NaCl stress, and shoot Na^+^ content and fresh weight in the presence of long-term NaCl stress (C). Data was accumulated from three cultivars of rice (*Oryza sativa* L.) grown under varying nutritional conditions. Inset: respective correlation analyses between ion content and fresh weight for root tissues.

**Figure 5:**
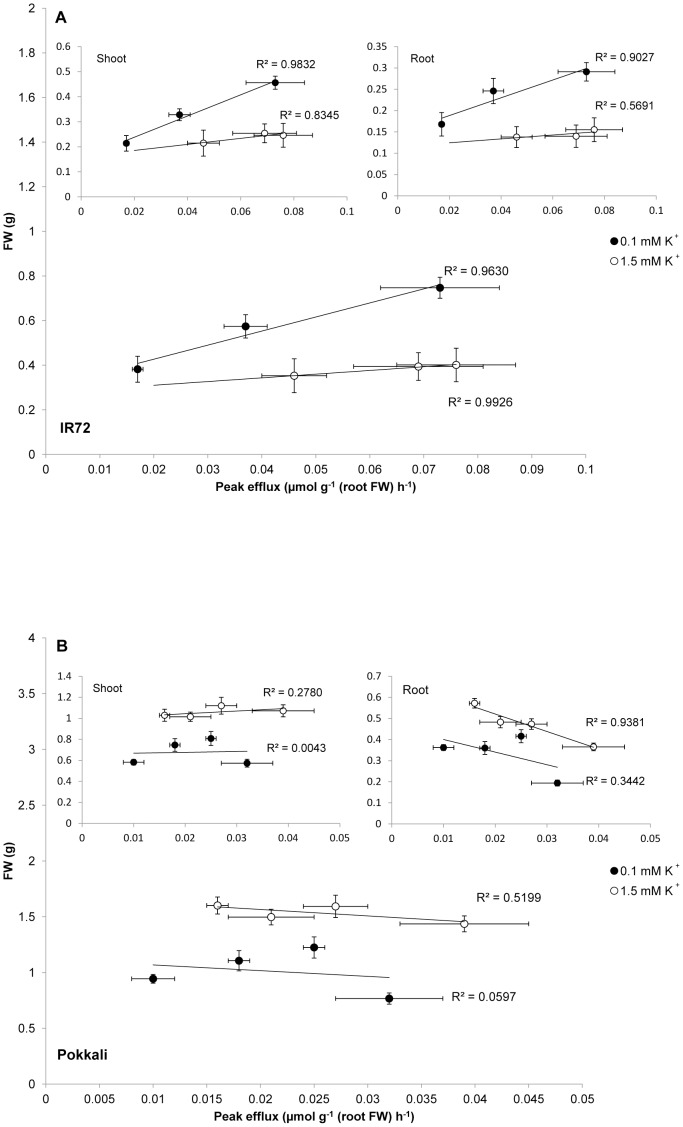
NaCl-stimulated K^+^ efflux and biomass. Correlation analyses between NaCl-stimulated peak K^+^ efflux from roots and total biomass (or shoot and root biomass, separately; insets) on long-term NaCl stress in (A) IR72 and (B) Pokkali under low and high K^+^ growth conditions. Axes labels for insets as in main figure. Error bars indicate ± SEM.

No measure of K^+^ status could predict plant performance either in the presence or absence of NaCl stress. When combining data from all cultivars and conditions, neither root nor shoot K^+^ content showed a correlation with FW in the absence (Fig. 4A) or presence (Fig. 4B) of long-term NaCl stress. Moreover, no general relationship was found between plant performance under long-term NaCl stress and the magnitudes of NaCl-stimulated peak K^+^ efflux, integrated K^+^ efflux or root K^+^ decline (Table S1). In fact, in only one scenario could a strong negative correlation (R^2^>0.34) be found between peak K^+^ efflux and tissue biomass under long-term NaCl stress (Fig. 5B, inset: roots at high K^+^). No correlations were found under low K^+^ conditions for Pokkali (Fig. 5B), and surprisingly, significant *positive* correlations were found for both shoot and root tissue for IR72 at both K^+^ levels (Fig. 5A).

In contrast to these findings with K^+^, shoot Na^+^ content showed a strong negative correlation (R^2^  =  0.77) with shoot biomass under long-term NaCl stress (Fig. 4C). This was not the case for root tissue (Fig. 4C, inset).

## Discussion

The present study is the first to examine NaCl-stimulated K^+^ efflux in rice and to relate this phenomenon to performance on long-term NaCl stress. Consistent with studies on other plant species (*e.g.*, barley [12], [20], wheat [23], bean [54], cotton [16], *Arabidopsis* [13], pea [55], alfalfa [56], and sunflower (our unpublished results)), we show that sudden exposure to high levels of NaCl produce a significant and sustained stimulation of K^+^ efflux in three cultivars of rice that differ dramatically in salt tolerance. We also show that this effect occurs regardless of nutritional history (Fig. 1), albeit to varying extents (Table 5). We should stress that this effect only occurs if NaCl concentrations are sufficiently high (*e.g.* 160 mM), as it was not observed in a lower range (25–75 mM; Fig. S1). By contrast, long-term exposure to 50 mM NaCl was sufficient to bring about toxicity in all cultivars, and in some cases even mortality (Tables 1, 2, 3, 4). These findings question the universal relevance of NaCl-stimulated K^+^ efflux to NaCl toxicity.

Recently, we investigated the mechanism underlying the efflux stimulation in barley roots, and concluded that membrane disintegrity due to osmotic and ionic effects was the cause [12], a conclusion that agreed with earlier explanations [15], [16], but opposed more recent explanations that attribute the effect to the gating of outwardly rectifying K^+^ channels by Na^+^-induced membrane depolarization [13]. In the present study in rice, we found that, as in barley, NaCl-stimulated K^+^ efflux showed no sensitivity to Cs^+^ (Fig. 2), an especially potent inhibitor of K^+^ fluxes [34], [57], [58], which discounts the involvement of outward-rectifying K^+^ channels. Simultaneous application of 160 mM NaCl with 10 mM Ca^2+^ showed significant suppressions of K^+^-efflux stimulation (Fig. 2). While Ca^2+^ is known to inhibit some ion channels [49]–[51], it is also well documented that calcium is critical to the stability of membranes including under NaCl stress [52], [59], [60], which may explain the suppression observed in the present study.

The agronomic importance of NaCl-stimulated K^+^ efflux has been suggested by the inverse relationship between the extent of efflux and the salt tolerance of wheat and barley cultivars, which thus may prove to be a valuable screening tool for some crops [20], [23], [61]. In our study, the salt-tolerant cultivar, Pokkali, did show significantly lower NaCl-stimulated K^+^ efflux compared to IR29 and IR72, under all conditions but one (Fig. 1). However, the stimulation of K^+^ efflux in IR72 did not fall between that of IR29 and Pokkali under most conditions (Fig. 1), even though IR72 clearly demonstrated intermediate sensitivity to long-term NaCl stress, in terms of survival, biomass decline, and shoot Na^+^ content (Tables 1, 2, 3, 4).

Nor was there a strong negative relationship observed between peak NaCl-stimulated K^+^ efflux and plant growth on NaCl, within the three cultivars of rice examined here, under varying nutritional conditions (Fig. 5). Within cultivars, only when correlation analyses were limited to a specific K^+^ level were any relationships observed. Even then, only one correlation was strongly negative for Pokkali (roots at high K^+^; Fig. 5B inset), while all correlations were in fact strongly positive for IR72 (Fig. 5A).

Long-term NaCl stress showed no correlation between peak (or integrated) efflux, and survival, biomass decline (both absolute and relative), tissue K^+^ content and its decline (both absolute and relative), and tissue Na^+^ content and its accumulation (both absolute and relative) (Table S1). Thus, it appears that in rice, NaCl-stimulated K^+^ efflux from the root system provides no utility in screening for performance under salinity stress.

Perhaps of greater surprise was the more fundamental observation that tissue K^+^ content showed no relationship with plant biomass in even the absence of salinity stress (Fig. 4; Table S1). It has long been known that ‘luxury consumption’ of K^+^ occurs when it is not nutritionally limiting [62]-[64]. Because plants can homeostatically maintain cytosolic [K^+^] at ∼100 mM, at the expense of vacuolar stores [65], they can maintain proper functioning against a background of widely varying tissue K^+^ levels. As shown in Table 1, these levels can be extremely low, as in the case of Pokkali at low K^+^, high NH_4_^+^, and 50 mM NaCl (18 and 40 µmol K^+^ g^-1^ FW in root and shoot, respectively), but can nevertheless be compatible with biomass that exceeds what is seen in other cultivars with much higher tissue K^+^ levels (*e.g.*, IR29 and IR72 at low K^+^, high NH_4_^+^, and without NaCl). Fig. 3 demonstrates that the loss of root K^+^ due to sudden NaCl exposure is relatively minor compared to the vast fluctuations in root K^+^ levels achieved by alterations in growth history in the absence of salt stress. Moreover, in some cases, it appears that increased K^+^ provision can in fact be detrimental to performance on long-term NaCl exposure. Except in plants grown on high NH_4_^+^, where it is clear that enhanced K^+^ availability is beneficial due to the alleviation of NH_4_^+^ toxicity [66], biomass decline due to long-term NaCl exposure was actually greater on high K^+^ in IR72 under low NH_4_^+^ and high NO_3_^−^ conditions. Furthermore, IR72 and IR29 did not survive at high K^+^ on low and high NO_3_^-^, respectively (Table 4). Thus, it becomes apparent that, at least in rice, focus on K^+^ status as a measure of plant performance under saline and non-saline conditions, is perhaps misguided.

By contrast, shoot Na^+^ content was a good predictor of biomass on long-term NaCl stress (R^2^  =  0.77; Fig. 4C). This is in good agreement with previous reports on rice that demonstrate strong negative correlations between shoot Na^+^ content and performance [67], [68]. Moreover, this was the only measure that displayed clear cultivar differences in the present work, based on salt tolerance, independent of growth history (*i.e.*, IR29>IR72>Pokkali; Tables 1, 2, 3, 4). It is believed that shoot Na^+^ accumulation in rice occurs preferentially via an apoplastic bypass pathway [69], [70], but is lower in salt-tolerant cultivars such as Pokkali [71]. It is also believed that elevated Ca^2+^ levels can reduce bypass flow of Na^+^ into the transpiration stream [72]. Indeed, under low-K^+^, high-Ca^2+^ (high-NO_3_^−^) conditions, all cultivars showed the lowest shoot Na^+^ content when grown on NaCl (Table 2). However, this was not observed at high K^+^, which may be related to the poorer performance on salinity with high K^+^, as mentioned above. Thus, it appears that monitoring K^+^ nutrition (including efflux and retention) in hopes of screening for salt tolerance in rice is not a promising strategy, and that focus should remain on shoot Na^+^ accumulation and the mechanisms by which it is brought about [22].

## Supporting Information

Table S1 **Correlation analyses between biomass, tissue content, and K^+^ efflux.** Pearson correlation matrix (R^2^ values) between measures of biomass, tissue K^+^/Na^+^ content, and K^+^ efflux, accumulated from three cultivars of rice (*Oryza sativa* L.) grown under varying nutritional conditions+/−long-term NaCl stress.
(PDF)

Figure S1 **Concentration dependence of NaCl-induced K^+^ efflux.** Response of K^+^ efflux from roots of intact rice (*Oryza sativa* L., cv. IR72) to sudden provision (at *t* = 15.5 min; see arrow) of varying concentrations of NaCl. Error bars indicate ± SEM.
(TIF)
