# Supplementary material for: K+ Efflux and Retention in Response to NaCl Stress Do Not Predict Salt Tolerance in Contrasting Genotypes of Rice (Oryza sativa L.)
Source: PLoS One. 2013 Feb 27;8(2):e57767. doi: 10.1371/journal.pone.0057767 (PMC3583904; doi:10.1371/journal.pone.0057767)
Supplement: Table S1 — Correlation analyses between biomass, tissue content, and K+ efflux. Pearson correlation matrix (R2 values) between measures of biomass, tissue K+/Na+ content, and K+ efflux, accumulated from three cultivars of rice (Oryza sativa L.) grown under varying nutritional conditions+/−long-term NaCl stress. (PDF) [file pone.0057767.s001.pdf]

A. Correlations (R^2) between measures of biomass, tissue K^+/Na^+ content, and K^+ efflux, accumulated from three cultivars of rice grown under varying nutritional conditions without NaCl stress.

		-NaCl																
		Shoot FW (g)	Root FW (g)	Shoot DW (g)	Root DW (g)	Shoot DW:FW	Root DW:FW	Shoot K^+ (µmol g^{-1} FW)	Root K^+ (µmol g^{-1} FW)	Shoot Na^+ (µmol g^{-1} FW)	Root Na^+ (µmol g^{-1} FW)	Shoot K^+:Na^+	Root K^+:Na^+	Root K^+ w/ ST[a] NaCl (µmol g^{-1} FW)	Root K^+ loss w/ ST NaCl (µmol g^{-1} FW)	Root K^+ loss w/ ST NaCl (% Ctrl)	Peak K^+ efflux (µmol g^{-1} FW h^{-1})	K^+_{cyt}[b] loss (µmol g^{-1} FW)
- NaCl	Shoot FW (g)	1.00	0.50	0.55	0.40	0.52	0.19	0.10	0.01	0.24	0.09	0.19	0.00	0.00	0.00	0.00	0.04	0.09
	Root FW (g)		1.00	0.09	0.97	0.30	0.55	0.27	0.23	0.33	0.07	0.38	0.04	0.23	0.03	0.18	0.19	0.25
	Shoot DW (g)			1.00	0.03	0.13	0.01	0.03	0.00	0.06	0.05	0.02	0.00	0.07	0.20	0.15	0.01	0.00
	Root DW (g)				1.00	0.22	0.64	0.18	0.18	0.30	0.03	0.33	0.01	0.21	0.09	0.23	0.25	0.31
	Shoot DW:FW					1.00	0.06	0.24	0.15	0.22	0.14	0.27	0.08	0.08	0.01	0.01	0.02	0.00
	Root DW:FW						1.00	0.09	0.06	0.49	0.12	0.33	0.01	0.08	0.28	0.31	0.14	0.27
	Shoot K^+ (µmol g^{-1} FW)							1.00	0.68	0.24	0.44	0.61	0.49	0.64	0.01	0.27	0.00	0.00
	Root K^+ (µmol g^{-1} FW)								1.00	0.20	0.24	0.53	0.57	0.93	0.05	0.15	0.02	0.00
	Shoot Na^+ (µmol g^{-1} FW)									1.00	0.49	0.69	0.23	0.21	0.09	0.25	0.01	0.04
	Root Na^+ (µmol g^{-1} FW)										1.00	0.43	0.59	0.17	0.00	0.11	0.09	0.02
	Shoot K^+:Na^+											1.00	0.48	0.60	0.03	0.38	0.05	0.05
	Root K^+:Na^+												1.00	0.50	0.03	0.08	0.01	0.02
	Root K^+ w/ ST NaCl (µmol g^{-1} FW)													1.00	0.00	0.36	0.09	0.01
	Root K^+ loss w/ ST NaCl (µmol g^{-1} FW)														1.00	0.58	0.16	0.03
	Root K^+ loss w/ ST NaCl (% Ctrl)															1.00	0.10	0.03
	Peak K^+ efflux (µmol g^{-1} FW h^{-1})																1.00	0.28
	K^+_{cyt} loss (µmol g^{-1} FW)																	1.00

[a] Short term (*i.e.*, 45 min); [b] cytosolic K^+ content.

B. Correlations (R^2) between measures of biomass, tissue K^+/Na^+ content, and K^+ efflux, accumulated from three cultivars of rice grown under varying nutritional conditions with and without NaCl stress.

		+NaCl																								
		Survival (Y/N)	Shoot FW (g)	Root FW (g)	Shoot DW (g)	Root DW (g)	Shoot DW:FW	Root DW:FW	Shoot FW decline (g)	Shoot FW decline (% Ctrl)	Root FW decline (g)	Root FW decline (% Ctrl)	Shoot K^+ (µmol g^{-1} FW)	Root K^+ (µmol g^{-1} FW)	Shoot K^+ loss (µmol g^{-1} FW)	Shoot K^+ loss (% Ctrl)	Root K^+ loss (µmol g^{-1} FW)	Root K^+ loss (% Ctrl)	Shoot Na^+ (µmol g^{-1} FW)	Root Na^+ (µmol g^{-1} FW)	Shoot Na^+ gain (µmol g^{-1} FW)	Shoot Na^+ gain (% Ctrl)	Root Na^+ gain (µmol g^{-1} FW)	Root Na^+ gain (% Ctrl)	Shoot K^+:Na^+	Root K^+:Na^+
- NaCl	Shoot FW (g)	0.36	0.66	0.43	0.66	0.19	0.40	0.14	0.43	0.07	0.01	0.10	0.13	0.03	0.39	0.29	0.00	0.01	0.44	0.33	0.44	0.36	0.35	0.33	0.20	0.40
	Root FW (g)	0.31	0.23	0.42	0.26	0.02	0.17	0.16	0.07	0.05	0.58	0.03	0.00	0.16	0.09	0.02	0.37	0.24	0.05	0.00	0.05	0.01	0.00	0.03	0.11	0.07
	Shoot DW (g)	0.11	0.12	0.04	0.12	0.03	0.02	0.01	0.54	0.05	0.01	0.02	0.06	0.00	0.10	0.08	0.08	0.07	0.06	0.32	0.06	0.07	0.33	0.25	0.00	0.25
	Root DW (g)	0.30	0.19	0.39	0.21	0.03	0.15	0.21	0.01	0.08	0.56	0.02	0.00	0.10	0.10	0.03	0.32	0.23	0.05	0.00	0.05	0.01	0.00	0.03	0.08	0.02
	Shoot DW:FW	0.21	0.56	0.32	0.44	0.13	0.74	0.00	0.01	0.30	0.01	0.12	0.00	0.25	0.07	0.04	0.05	0.01	0.37	0.04	0.36	0.32	0.05	0.13	0.49	0.24
	Root DW:FW	0.32	0.05	0.21	0.09	0.08	0.00	0.51	0.09	0.17	0.11	0.03	0.07	0.00	0.12	0.10	0.05	0.10	0.03	0.00	0.03	0.00	0.00	0.00	0.02	0.02
	Shoot K^+ (µmol g^{-1} FW)	0.01	0.07	0.15	0.12	0.27	0.04	0.00	0.00	0.02	0.07	0.02	0.37	0.34	0.01	0.06	0.47	0.44	0.00	0.05	0.00	0.00	0.06	0.10	0.23	0.10
	Root K^+ (µmol g^{-1} FW)	0.00	0.03	0.09	0.03	0.09	0.03	0.01	0.01	0.03	0.27	0.13	0.48	0.69	0.07	0.22	0.90	0.39	0.02	0.10	0.02	0.05	0.10	0.11	0.24	0.09
	Shoot Na^+ (µmol g^{-1} FW)	0.27	0.16	0.25	0.20	0.20	0.01	0.45	0.03	0.24	0.02	0.07	0.01	0.04	0.01	0.02	0.11	0.02	0.03	0.01	0.03	0.01	0.00	0.00	0.11	0.02
	Root Na^+ (µmol g^{-1} FW)	0.05	0.04	0.10	0.00	0.24	0.20	0.01	0.04	0.06	0.01	0.02	0.05	0.00	0.01	0.04	0.00	0.01	0.04	0.02	0.04	0.04	0.00	0.23	0.00	0.02
	Shoot K^+:Na^+	0.10	0.22	0.41	0.32	0.01	0.07	0.18	0.01	0.22	0.04	0.02	0.17	0.38	0.01	0.01	0.48	0.27	0.04	0.01	0.04	0.00	0.01	0.04	0.47	0.14
	Root K^+:Na^+	0.01	0.00	0.00	0.01	0.20	0.02	0.02	0.00	0.00	0.03	0.06	0.32	0.38	0.05	0.16	0.36	0.15	0.03	0.00	0.03	0.05	0.01	0.23	0.16	0.17
	Root K^+ w/ ST[a] NaCl (µmol g^{-1} FW)	0.01	0.04	0.13	0.05	0.07	0.05	0.00	0.07	0.09	0.28	0.08	0.37	0.50	0.03	0.14	0.90	0.50	0.01	0.18	0.01	0.04	0.18	0.16	0.22	0.02
	Root K^+ loss w/ ST NaCl (µmol g^{-1} FW)	0.03	0.01	0.03	0.02	0.01	0.02	0.23	0.21	0.18	0.00	0.05	0.08	0.17	0.12	0.12	0.00	0.07	0.02	0.14	0.02	0.00	0.14	0.07	0.00	0.34
	Root K^+ loss w/ ST NaCl (% Ctrl)	0.02	0.01	0.07	0.03	0.05	0.01	0.14	0.17	0.14	0.08	0.00	0.03	0.00	0.03	0.00	0.21	0.41	0.00	0.26	0.00	0.01	0.28	0.24	0.01	0.14
	Peak K^+ efflux (µmol g^{-1} FW h^{-1})	0.00	0.27	0.34	0.24	0.25	0.25	0.29	0.00	0.20	0.19	0.01	0.05	0.01	0.10	0.10	0.15	0.11	0.09	0.01	0.08	0.02	0.00	0.04	0.10	0.00
	K^+_{cyt}[b] loss (µmol g^{-1} FW)	0.04	0.17	0.39	0.24	0.15	0.03	0.44	0.00	0.14	0.12	0.05	0.05	0.03	0.10	0.07	0.01	0.00	0.14	0.10	0.13	0.06	0.11	0.11	0.08	0.09

[a] Short term (*i.e.* , 45 min); [b] cytosolic K^+ content.

C. Correlations (R^2) between measures of biomass and tissue K^+/Na^+ content, accumulated from three cultivars of rice grown under varying nutritional conditions with NaCl stress.

		+NaCl																							
		**Shoot FW (g)**	**Root FW (g)**	**Shoot DW (g)**	**Root DW (g)**	**Shoot DW:FW**	**Root DW:FW**	**Shoot FW decline (g)**	**Shoot FW decline (% Ctrl)**	**Root FW decline (g)**	**Root FW decline (% Ctrl)**	**Shoot K^+ (µmol g^{-1} FW)**	**Root K^+ (µmol g^{-1} FW)**	**Shoot K^+ loss (µmol g^{-1} FW)**	**Shoot K^+ loss (% Ctrl)**	**Root K^+ loss (µmol g^{-1} FW)**	**Root K^+ loss (% Ctrl)**	**Shoot Na^+ (µmol g^{-1} FW)**	**Root Na^+ (µmol g^{-1} FW)**	**Shoot Na^+ gain (µmol g^{-1} FW)**	**Shoot Na^+ gain (% Ctrl)**	**Root Na^+ gain (µmol g^{-1} FW)**	**Root Na^+ gain (% Ctrl)**	**Shoot $K^+:Na^+$**	**Root $K^+:Na^+$**
**+NaCl**	**Shoot FW (g)**	1.00	0.82	0.95	0.40	0.75	0.33	0.01	0.60	0.02	0.44	0.16	0.12	0.51	0.39	0.00	0.01	0.77	0.22	0.77	0.61	0.25	0.30	0.55	0.37
	**Root FW (g)**		1.00	0.86	0.30	0.53	0.51	0.00	0.61	0.00	0.38	0.07	0.16	0.44	0.28	0.04	0.00	0.62	0.12	0.62	0.45	0.15	0.24	0.55	0.31
	**Shoot DW (g)**			1.00	0.30	0.58	0.45	0.02	0.55	0.01	0.45	0.13	0.09	0.56	0.38	0.01	0.00	0.74	0.23	0.74	0.56	0.25	0.24	0.55	0.39
	**Root DW (g)**				1.00	0.32	0.31	0.01	0.37	0.09	0.48	0.60	0.01	0.28	0.50	0.13	0.29	0.49	0.23	0.48	0.35	0.30	0.52	0.08	0.06
	**Shoot DW:FW**					1.00	0.07	0.00	0.52	0.01	0.26	0.10	0.10	0.33	0.26	0.01	0.01	0.62	0.04	0.62	0.59	0.07	0.27	0.42	0.14
	**Root DW:FW**						1.00	0.01	0.35	0.01	0.40	0.27	0.01	0.47	0.43	0.01	0.00	0.31	0.09	0.30	0.14	0.10	0.11	0.11	0.03
	**Shoot FW decline (g)**							1.00	0.29	0.14	0.10	0.01	0.02	0.02	0.01	0.01	0.00	0.00	0.14	0.00	0.00	0.13	0.08	0.04	0.08
	**Shoot FW decline (% Ctrl)**								1.00	0.13	0.62	0.10	0.09	0.27	0.23	0.00	0.02	0.58	0.03	0.57	0.43	0.04	0.08	0.53	0.09
	**Root FW decline (g)**									1.00	0.54	0.10	0.03	0.03	0.07	0.39	0.36	0.15	0.10	0.15	0.17	0.09	0.03	0.04	0.01
	**Root FW decline (% Ctrl)**										1.00	0.38	0.00	0.42	0.48	0.23	0.27	0.70	0.25	0.70	0.60	0.27	0.28	0.29	0.11
	**Shoot K^+ (µmol g^{-1} FW)**											1.00	0.30	0.55	0.84	0.46	0.30	0.39	0.28	0.39	0.35	0.32	0.44	0.00	0.00
	**Root K^+ (µmol g^{-1} FW)**												1.00	0.04	0.13	0.37	0.02	0.02	0.02	0.02	0.02	0.02	0.00	0.45	0.46
	**Shoot K^+ loss (µmol g^{-1} FW)**													1.00	0.89	0.07	0.02	0.65	0.23	0.65	0.59	0.25	0.31	0.13	0.08
	**Shoot K^+ loss (% Ctrl)**														1.00	0.21	0.11	0.61	0.27	0.61	0.54	0.31	0.42	0.05	0.02
	**Root K^+ loss (µmol g^{-1} FW)**															1.00	0.67	0.09	0.27	0.10	0.15	0.27	0.24	0.09	0.00
	**Root K^+ loss (% Ctrl)**																1.00	0.18	0.46	0.18	0.22	0.49	0.44	0.00	0.10
	**Shoot Na^+ (µmol g^{-1} FW)**																	1.00	0.41	1.00	0.92	0.45	0.53	0.45	0.31
	**Root Na^+ (µmol g^{-1} FW)**																		1.00	0.41	0.39	0.98	0.59	0.09	0.54
	**Shoot Na^+ gain (µmol g^{-1} FW)**																			1.00	0.93	0.45	0.53	0.45	0.31
	**Shoot Na^+ gain (% Ctrl)**																				1.00	0.43	0.55	0.38	0.30
	**Root Na^+ gain (µmol g^{-1} FW)**																					1.00	0.71	0.09	0.52
	**Root Na^+ gain (% Ctrl)**																						1.00	0.07	0.28
	**Shoot $K^+:Na^+$**																							1.00	0.41
	**Root $K^+:Na^+$**																								1.00
