# Supplementary figures and images for: K+ Efflux and Retention in Response to NaCl Stress Do Not Predict Salt Tolerance in Contrasting Genotypes of Rice (Oryza sativa L.)
Source: PLoS One. 2013 Feb 27;8(2):e57767. doi: 10.1371/journal.pone.0057767 (PMC3583904; doi:10.1371/journal.pone.0057767)

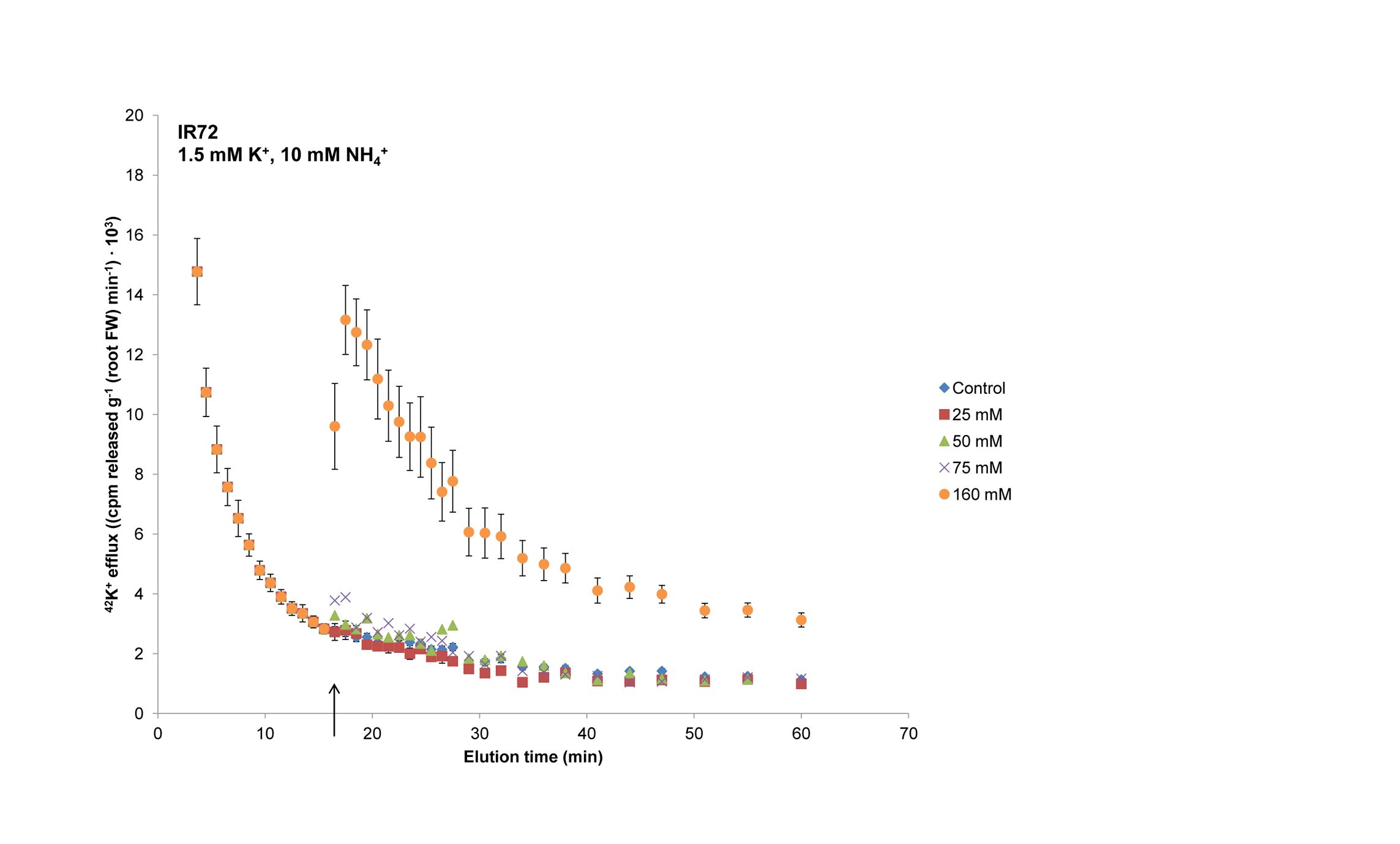

Supplement: Figure S1 — Concentration dependence of NaCl-induced K+ efflux. Response of K+ efflux from roots of intact rice (Oryza sativa L., cv. IR72) to sudden provision (at t = 15.5 min; see arrow) of varying concentrations of NaCl. Error bars indicate ± SEM. (TIF) [file pone.0057767.s002.tif]
